# Investigation of rheological, physicochemical, and sensorial properties of traditional low‐fat Doogh formulated

**DOI:** 10.1002/fsn3.3647

**Published:** 2023-08-31

**Authors:** Fatemeh Rahmati, Abbas Mahjoorian, Fatemeh Fazeli, Sharagim Ranjbar

**Affiliations:** ^1^ Department of Food Science & Technology, Ayatollah Amoli Branch Islamic Azad University Amol Iran; ^2^ Department of Food Hygiene, Faculty of Veterinary medicine, Tabriz Medical Science Islamic Azad University Tabriz Iran

**Keywords:** activation energy, Arrhenius equation, Caspian Sea *Huso huso*, Doogh, *Ostwald‐de Waele* model

## Abstract

Doogh is a fermented beverage made from yoghurt with water and salt. Similarly, drinks based on yoghurt are available in different countries with varying degrees of dilution, fat content, rheological properties, and taste. In this project, the use of mathematical calculations in describing rheological parameters from traditional low‐fat Doogh enriched with Caspian Sea (*Huso huso*) gelatin (0.4 w/v %), xanthan hydrocolloids (0.4 w/v %), and their mixture at a ratio of 0.2:0.2 w/v % studied. Also, serum isolation, pH, and sensory evaluation of samples were investigated. Also, the relationship between apparent viscosity and temperature of Doogh samples using the Arrhenius equation was studied. The sensory evaluation revealed that the overall acceptance scores of the samples containing gelatin, xanthan, mix, and control were 4.31, 4.33, 4.58, and 4.12, respectively. The study on serum separation value showed control sample (45.07) and mix sample (0.84) at the end of 30 days. On the first day, the pH of the Doogh samples decreased with the addition of hydrocolloids, and this trend was time dependent. pH reduction was higher in Doogh with gelatin than in other samples. Mathematical calculations showed that the low‐fat Doogh is a non‐Newtonian type and shear‐thinning (Pseudoplastic) fluid. The activation energy was calculated between 11.65 and 19.15 kJ/mol. According to the obtained results, it concluded that the use of two hydrocolloid compounds improved the physicochemical and sensory characteristics of the low‐fat Doogh samples. Also, the *Ostwald*‐*de Waele* mathematical model had a high correlation with the rheological behavior of the samples.

## Graphical abstract

Two‐phase Doogh is the main challenge of keeping it in the refrigerator. Use of hydrocolloids for the stability of dairy drinks. Application of mathematical models in estimating rheological models of Doogh samples.

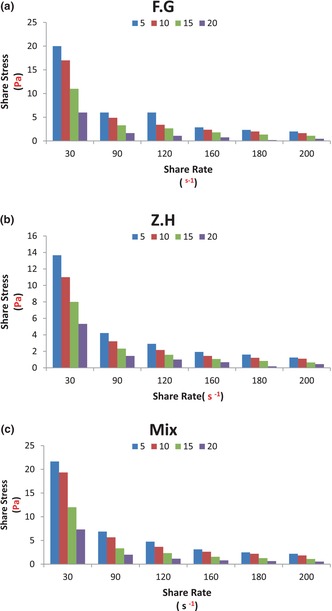

## 1 INTRODUCTION

Doogh is one of the traditional drinks in Iran and some other countries, such as Eastern Europe, the Middle East, and Asia (Sani et al., 2019). This product is obtained by diluting yoghurt with drinking water, fermented whey, or buttermilk. Also, edible aromatic herbs like mint, clove, and coriander, or their natural essential oils, can be used in Doogh formulation (Azarikia & Abbasi, 2010). One of the most significant challenges in the production of dairy‐based beverages such as Doogh is phase separation (Turkmen et al., 2019). This phenomenon occurs due to the separation of the serum‐water phase in the Doogh structure, which leads to reduced consumer appeal and changes in texture and color of the product (McCain et al., 2018). Several different parameters can contribute to the occurrence of serum separation of Doogh (Azarikia & Abbasi, 2010). The most important factors include the use of inappropriate raw materials, long‐term storage, microbial growth, high storage temperatures, and the absence of thickening agents such as hydrocolloids (Imeson, 2011). In fact, these compounds create complexes between the water and protein–carbohydrate chains, which enhance water retention in that structure and delay phase separation (Liang & Luo, 2020).

Hydrocolloids are widely utilized in the food industry. These compounds are consumed as condensing and gelling agents, stabilizing foams in emulsions, solutions, emulsifiers, and preventing the formation of ice and sugar crystals, barrier properties against gas/moisture, preventing moisture loss, enhancing moisture retention, and also moisture retention (Doublier et al., 2017; Li & Nie, 2016; Williams & Phillips, 2021). Nowadays, most researchers recommend that the addition of stabilizers or hydrocolloid compounds as a practical solution to avoid serum separation in acidic dairy beverages acts in two ways: (a) either as a thickening agent such as locust bean gum, alginate, xanthan, and guar or (b) anionic hydrocolloids such as gelatin, pectin, gellan, tragacanth, Lambda carrageenan, and carboxymethyl cellulose (CMC) that directly interact with other components in dairy products (Keller, 2020; Saha & hattacharya, 2010; Everett & McLeod, 2005; BeMiller, 2008).

Xanthan gum is an extracellular polysaccharide secreted by *Xanthomonas campestris* microorganism. The main branch is d‐glucose units with beta (1–6) bonds which are attached at the position of three glucose molecules to a trisaccharide chain consisting of two D‐mannose units and one d‐galacturonic acid unit. Xanthan is soluble in cold water and its viscosity is very stable over a wide range of temperatures and pH. Xanthan gum solutions show lubricating behavior due to their viscosity reduction (Bak & Yoo, 2018; Garcıa‐Ochoa et al., 2000; Palaniraj & Jayaraman, 2011; Sworn, 2021). Dario et al. (2011) reported that calcium chloride and calcium nitrate at concentrations of 1 and 10 g L^−1^ can decrease the solubility of xanthan solutions (Dario et al., 2011).

On the other hand, gelatin is one of the most important biopolymers due to its favorable physicochemical and rheological properties (Huang et al., 2019). This ingredient is widely used in the food industry, including in the production of yoghurt, desserts, pastries, and bread. In recent years, the demand for gelatin and collagen in various industries has increased significantly (Mariod & Fadul, 2013). Due to the high percentage of collagen in pigs and bovine, major producers in the world use it as a raw material for gelatin production. However, for various sociocultural reasons, alternative sources are increasingly in demand. Fish gelatin differs from mammals' in some respects. Generally, fish gelatins have lower viscosity and melting point compared to mammalian sources (Mahjoorian et al., 2021). There are four sturgeon species in the Caspian Sea basin (which is bordered by Azerbaijan, Iran, Kazakhstan, Turkmenistan, and the Russian Federation), including *Acipenser gueldenstaedtii*, *A. persicus*, *A. stellatus*, and *Huso huso*. *Huso huso* is a warm water fish belonging to the *Acipenseridae* family. Annually, large quantities of *Huso huso* meat fish are consumed in Iran, and the skin is discarded. This skin is a good source of collagen, which can be used for gelatin extraction.

It is also important to note that the rheological behavior of liquids (beverages) is important in many parts of industrial plants, such as pipeline design and pump power determination (Tucker, 2017; Vélez‐Ruiz, 2019; Zheng, 2019). Besides, product stability during storage (which is effective on oral sensation) is a key factor in quality that can affect the rheological behavior of product (Stokes et al., 2013). Therefore, in view of the above, the importance of this research seems necessary. Azarikia and Abbasi (2010) investigated the effect of three Iranian tragacanth species on the rheological characteristics and stability of nonfat Doogh. The highest stability was observed in samples containing Astragalus gossypinus gum with 0.3% concentration. On the other hand, the apparent viscosity of the Doogh sample containing this species has higher viscosity than that of the other species, and its sensitivity to frequency increase was very low, which is very important in the industry (Azarikia & Abbasi, 2010). Mahjoorian et al. (2020) pointed out that the power–low model had the best fit with the gelatin solution of skin gelatin extracted from the Caspian Sea Huso huso at different concentrations and temperatures. This study aimed to evaluate the effect of gelatin extracted from the skin of Caspian Sea *Huso huso* and xanthan gum including single and double forms on rheological properties, pH, serum separation, and sensory evaluation of Doogh samples. Also, the apparent viscosity dependency is predicted by temperature based on the Arrhenius equation.

## 2 MATERIALS AND METHODS

### 2.1 Material

Xanthan gum was purchased from Sigma Aldrich. Also, milk containing 0.5% fat was provided by the Kalleh Dairy Co, Mazandaran province. Table 1 shows some of the chemical compositions of milk used in yoghurt production. Yoghurt starter *CH1* was obtained from Christian Hansen Denmark. The skin of the Caspian Sea *Huso huso* was transferred to the laboratory by Kian Mahi Khazar Co. The skin was kept in a freezer at −18°^C^. Chemical compounds (acetic acid and sodium hydroxide) used in this study were purchased from Merck. All reagents and chemical solutions were of analytical grade.

**TABLE 1:** Chemical composition of milk used in yoghurt production.

pH	Moisture (%)	Ash (%)	Fat (%)	Carbohydrate (%)	Protein (%)
6.62 ± 0.021	88.35 ± 0.075	0.48 ± 0.038	1.54 ± 0.015	12.64 ± 0.076	8.38 ± 0.044

### 2.2 Extraction of gelatin from Caspian Sea *Huso huso*

The frozen skin of Caspian Sea *Huso huso* was thawed at 7°C for 20 h and then cut into small pieces of 3 cm (wide) × 4 cm (long) using scissors. The pieces were washed using cold tap water (at 4°C) for 10 min with a water/skin ratio of 6:1 (w/w). Washing was performed three times. Then, they were placed on the textile for 5 min to remove excess water. Gelatin extracted using the method proposed by Mahjoorian et al. (2020) with a slight modification. Fifty grams of defrosted and washed skin was submerged in a beaker containing different concentrations of sodium hydroxide solution (0.2 N) in a refrigerator at 7°C with a skin/solution ratio of 1:5 (w/v) to remove any noncollagenous proteins. The mixture was stirred for 3 h at room temperature (25°C) using a magnetic stirrer. The sodium hydroxide solution was changed every hour for three times. The residues were washed with tap water until a neutral or faintly basic pH was obtained. Washing the residues with tap water continued to reach an almost neutral pH. In the next step, the residues were mixed with an acetic acid solution (2 N) at a skin/solution ratio of 1:5 (w/v) in order to swell the collagenous ingredient in the Caspian Sea *Huso huso* skin matrix. The mixture was stirred at room temperature (25°C) with a magnetic stirrer for 2 h. After the blending operations were complete, the skin was washed with tap water until a neutral pH was obtained. Finally, in order to proceed with gelatin extraction, the swollen skin was mixed with distilled water at 45−55°C with a ratio of 1:10 (w/v) for 16–18 h. The solution's temperature was adjusted using a water bath (Memmert, WB14). At the allocated time, the mixtures were filtered using a Buchner funnel with a Whatman No.4 filter paper (Whatman International, Ltd.). Then, the filtrates were dried using a vacuum dryer (Memmert, VO400) at 40°C and 180 mbar. Gelatin powder was made immediately after the extraction or within a maximum of 24 h with the extracted material kept in a refrigerator at 7°C and it was subsequently subjected to analyses (Mahjoorian et al., 2020). The composition, characterization, and functional properties of the obtained gelatin from the Caspian Sea *Huso huso* skin are shown in Table 2.

**TABLE 2:** Composition, characterization, and functional properties of the obtained gelatin from Caspian Sea (*Huso huso*) skin.

Dry matter (%)	Water solubility (%)	Moisture (%)	Melting point (°C)	Yield (%)	Viscosity (mPa.s)	Protein (%)
91.42 ± 0.076	91.08 ± 0.005	8.63 ± 0.042	23.52 ± 0.01	22.45 ± 0.073	4.16 ± 0.041	85.38 ± 0.046

### 2.3 Preparation of traditional low‐fat yoghurt

Low‐fat fresh milk (0.5%) was prepared from the Kalleh Dairy Company, then homogenized in the laboratory by a homogenizer (AVP) in a two‐step homogenization operation (140 and 60 bar) and in a viscobator for 15 min at 90°C. Samples were subjected to heat treatment, then cooled to 40°C, and inoculated at the same temperature with a starter culture of yoghurt starter including *Lactobacillus delbrueckii* subspecies Bulgaricus (*Lactobacillus delbrueckii* subsp. Bulgaricus) and *Streptococcus thermophilus* (*Streptococcus thermophilus*) at 42°C until samples acidity reach to pH about 4.32. Finally, the samples were stored at 5°C.

### 2.4 Preparation of traditional low‐fat Doogh

For Doogh production, 40% w/w low‐fat yoghurt (0.5%), 59.3 w/w, distilled water, 0.7 w/w% salt with 95% purity, xanthan, and gelatin hydrocolloid were used. Fish gelatin (0.4 w/v %), xanthan (0.4 w/v %), Mix (0.2:0:2 w/v %), and salt dissolved in distilled water, subsequently added to the mixture. One‐step homogenization (150 bar) was performed carefully, and then the mixtures were heat‐treated in a viscobator at 85°C for 1 min. Doogh samples were filled in plastic bottles after reaching 14°C and stored in the refrigerator at 5°C. Table 3 shows the chemical composition of Doogh samples.

**TABLE 3:** The chemical composition of Doogh.

Ash (%)	Protein (%)	Energy (Cal)	pH	Zn (mg/100)	Na (mg/100)	Cu (mg/100)	Calcium (mg/100)	Carbohydrate (%)	Fat (%)
0.91 ± 0.044	2.15 ± 0.027	51.9 ± 0.065	4.35 ± 0.033	0.84 ± 0.016	316 ± 0.078	0.38 ± 0.012	65.15 ± 0.028	4.6 ± 0.019	1.50 ± 0.023

### 2.5 Rheological test

In this study, we used VMR rotary viscometer (Viscotech) to measure the viscosity of Doogh samples. Rheological tests were performed at six temperature levels of 5, 10, 15, 20, 25, and 30°C and different shear rates 0, 10, 20, 30, 40, 50, 60, 70, 80, 90, 100, and 200 rpm (s^−1^) in the presence of three different hydrocolloids types (gelatin, xanthan, and the combination of the two hydrocolloids) by a single cylindrical rotary viscometer. As VMR rotary viscometer viscosity decreases with increasing temperature, R2–R5 spindles at different temperatures were used for greater accuracy in measuring the viscosity of the samples. The apparent viscosity of Doogh samples was measured at 20 points over a relatively short period (1 min) throughout 0–200 rpm. Excel software was used to fit the data.

### 2.6 Modeling

In order to model the rheological behavior of low‐fat Doogh samples, apparent viscosity (*η*) and rotational velocity (rpm) were recorded by a viscometer. Assuming that the low‐fat Doogh containing hydrocolloid follows the power law relation (Relation 1), the stability factor (*K*) and the flow behavior index (*n*) can be approximated as follows.

(1) $$\tau =k\dot{\gamma^{n}},$$

The apparent viscosity is obtained from the following relation:

(2) $$\mu =\frac{\tau }{\dot{\gamma }}=\frac{k\dot{\gamma^{n}}}{\dot{\gamma }}=k{\dot{\gamma }}^{n-1}.$$

The shear rate (*s* ^−1^) is as follows for a power–law fluid in a rotational viscometer [16].

(3) $$\dot{\gamma }=\frac{2\omega }{n}=\frac{4\mathrm{\pi N}}{n}.$$

By taking the natural logarithm of the parties to the relationship (2) the following:

(4) $$\ln\mu =\ln K-\left(n-1\right)\ln n+\left(n+1\right)\ln\;\left(4\mathrm{\pi N}\right).$$

In these equations, *τ* is the shear stress (Pa), *K* is the stability factor (Pa.s), *s* ^−1^ is the shear rate, *n* is the flow behavior index (without dimension), *η* is the apparent viscosity (Pa.s), *ω* is the rotational speed (Hz) or (N/s), and *N* is the rotational speed of the spindle (rpm).

Considering *Y* = ln*η* and *X* = ln (4*πN*) and plotting *Y* according to *X*, the rheological parameters of low‐fat Doogh using linear regression through slope (*n* – 1) and width of origin (ln*K*– (*n*–) 1) lnn) is the equivalent of a computable line. In these relationships, *N* is the spindle rotation speed in terms of seconds per second. By substituting the values of flow behavior index and stability factor in Equation (1), the amount of shear stress (*τ*) can be calculated for each shear rate. It should be noted that the values of the shear rate are determined by Equation (3).

### 2.7 Effect of temperature on viscosity

The effect of temperature on the gelatin viscosity, *μ* is described by the Arrhenius relationship as follows:

(5) $$\mu ={\mu_{o}}_{T}\exp\left(\frac{E_{a}}{\mathrm{RT}}\right),$$

where *μ* is the gelatin viscosity (Pa.s), *μ*_*T*_ is the pre‐exponential constant (Pa.s), *E*_*a*_ is the activation energy (kJ/mol), *R* is the universal gas constant (8.314 × 10^−3^ kJ/mol.K), and *T* is the absolute temperature (*K*).

### 2.8 Serum separation

After pouring the samples into the graduated cylinder, the capping was done with aluminum foil. The Doogh samples were transferred to the refrigerator and stored at 4°C for 30 days. The volume of product from the end of the graduated cylinder to the next phase separation (mL) was reported at specified intervals 7 days after production.

### 2.9 Sensory evaluation

The sensory evaluation test was performed by a group of 15 trained panelists in the age range of 20–40 years (male and female). All panelists conducted using a 5‐point hedonic scoring method. Questionnaires were prepared and distributed to the evaluation team. The aroma, taste, firmness, color, and total acceptability parameters were determined.

### 2.10 Statistical analysis

In order to analyze the quantitative values of physicochemical, rheological, and organoleptic tests of samples, the SPSS software SPSS 22.0 for Windows, SPSS Inc. was used. After controlling data normality through Kolmogorov–Smirnov test, the values were analyzed with one‐way ANOVA to determine the significant difference. In cases where the overall effect of treatments was found to be significant, LCD test at 95% probability level was used to compare the mean values. Three replications were evaluated for each sample.

## 3 RESULTS AND DISCUSSIONS

**FIGURE 1:**
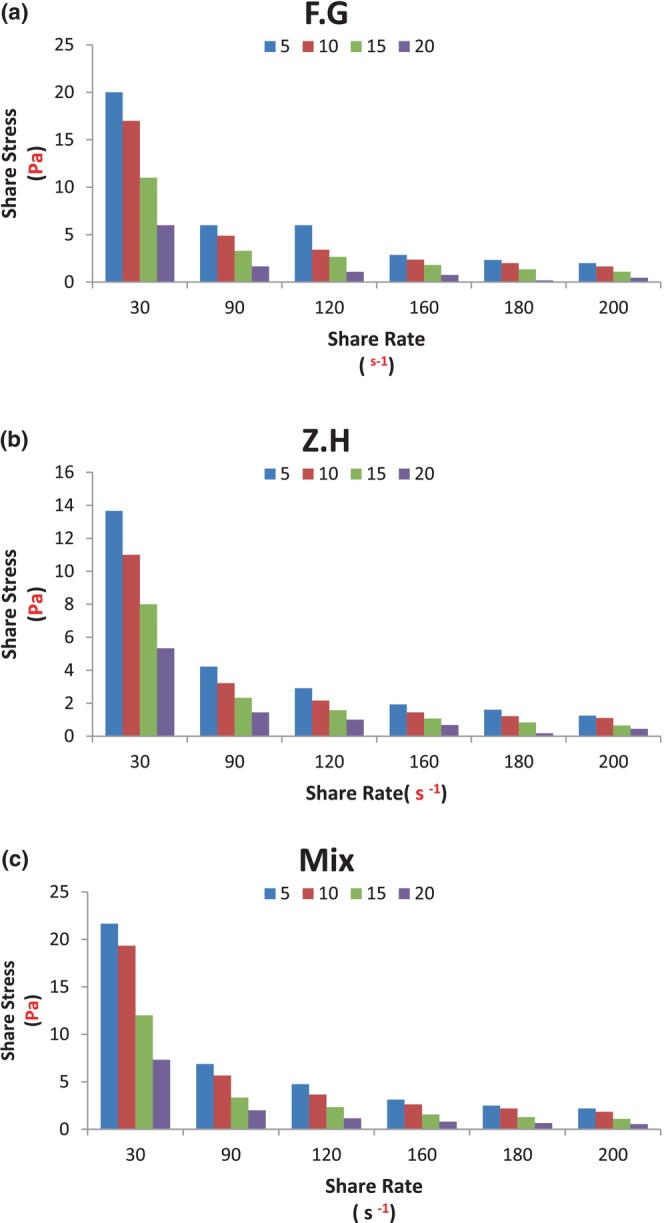
Influence of temperature‐type hydrocolloid composition on the shear stress–shear rate changes curves of traditional low‐fat Doogh: (a) gelatin, (b) xanthan, and (c) gelatin + xanthan.

Figure 1a–c shows the shear stress versus shear rates for low‐fat Doogh samples formulated with gelatin and xanthan hydrocolloids at different concentrations and temperatures.

As seen, considering the same temperature and constant shear rates, the Doogh sample enriched with gelatin has higher shear stresses compared to samples containing xanthan gum and Mix (gelatin/xanthan), which indicates the synergistic effect of hydrocolloids in Mix sample. To elucidate this argument, shear stress and apparent viscosity values at a shear rate and constant temperature (50 s − ^1^ and 5°C) for low‐fat Doogh for different hydrocolloids were predicted by the *Ostwald*‐*de* Waele model. The relationships between shear rate and shear stress due to the effect of the type of hydrocolloid used in the low‐fat Doogh formulation were fitted to the rheological properties based on the *Ostwald*‐*de Waele* model (power law) and the results are shown in Table 4. Jouki et al. (2014) point out that the type of hydrocolloid considerably affected the apparent viscosity due to the difference in the constituent and structure of the gel (Jouki et al., 2014). As seen, the rheological properties of the Doogh samples were different, so in samples prepared with different hydrocolloids, increasing the storage temperature changes the rheological behavior from mild (or nearly Newtonian) dilution in the samples stored at 5°C to more non‐Newtonian diluting behavior (Pseudoplastic) stored in Doogh samples at 30°C. As can be seen, the processing conditions and types of hydrocolloid compounds play a key role in the rheological properties of foods (Culetu et al., 2021).

**TABLE 4:** Percentage increase values of shear stress and apparent viscosity in shear rate 50 s^‐1^ and temperature 5°C.

	Type				Percentage increase
		Shear stress	Apparent viscosity	Shear stress	Apparent viscosity
		(Pa)	(mPa.s)	(Pa)	(mPa.s)
F.G		17.45	387	129.13	133
Xanthan		14.38	276	104	100
Mix		19.62	394	137.78	138

Doogh is considered as a shear‐thinning fluid. The relationship between shear rate and apparent viscosity is nonlinear, so Doogh is a non‐Newtonian fluid (Karim et al., 2017). The results showed that shear stress and apparent viscosity were significantly higher in fish gelatin than in xanthan. On the other hand, the synergistic effect of Mix (gelatin: xanthan) was more than xanthan sample. The reason for this phenomenon is referred to the presence of combination of hydrocolloids (fish gelatin/xanthan) could be due to the high amount of dry matter and the increased internal interaction between the particles in Doogh (Karim et al., 2017). Considering xanthan as the control sample and assuming 100% shear stress and apparent viscosity, the percentage of increasing shear stress and apparent viscosity was calculated for gelatin and (gelatin: xanthan) Mix samples. The percentage of increase in the above hydrocolloids relative to the xanthan was calculated as 129.13 and 137.78% for shear stress and 113 and 138 for apparent viscosity, respectively. The results related to the rheological behavior were similar to other studies when hydrocolloids were used in acidic milk beverages (Khanniri et al., 2019).

**FIGURE 2:**
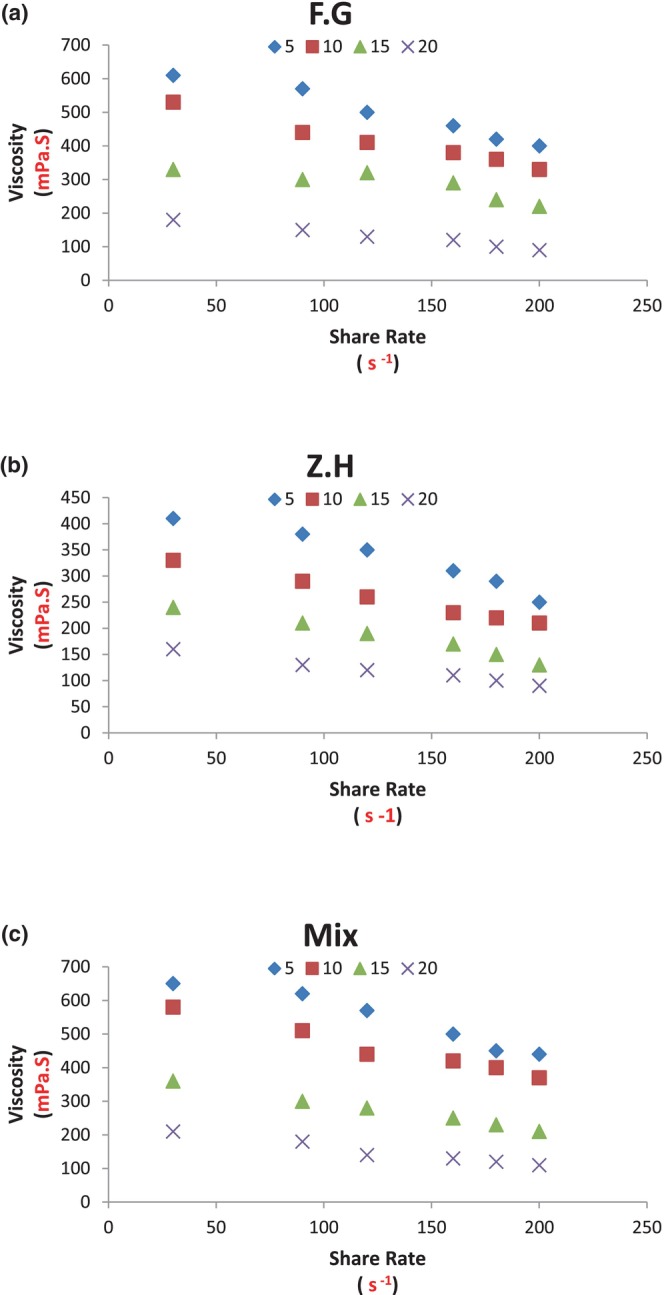
Influence of temperature‐type hydrocolloid composition on the curve of apparent viscosity–shear rate variations of traditional low‐fat Doogh: (a) gelatin, (b) xanthan, and (c) gelatin + xanthan.

Figure 2a–c shows the apparent viscosity versus shear rate curves of Doogh samples prepared with different hydrocolloids and temperatures. The Doogh samples prepared with gelatin hydrocolloid and Mix (gelatin: xanthan) at low shear rates have a much higher apparent viscosity, and with the increasing shear rate, the apparent viscosity decrease observed in all samples, which indicates a shear‐thinning behavior fluid (Doogh). The remarkable point was that in all samples containing hydrocolloids, there was a sudden decrease in apparent viscosity at low shear rates, whereas after this sudden decrease, the apparent viscosity of the samples decreased with a lower slope. This behavior can be attributed to the decrease in particle size at high shear rates (Da Cruz et al., 2002; Koos et al., 2014; Song et al., 2013). The results also showed that increasing the temperature decreased the apparent viscosity. Regarding the decrease in viscosity with temperature, it can be pointed out that in liquids the main factor of viscosity is the intermolecular bonding or in other words the intermolecular absorption force of the particles which prevents their free movement. As the temperature increases, the distance between the molecules increases, so the intermolecular adsorption, similarly the viscosity decreases (Severa et al., 2010).

The values of flow behavior index (*n*) for the different types of Doogh samples produced at different storage temperatures are reported in Table 5. As can be seen, at different storage temperatures, the highest value of flow behavior index was for Mix sample, while the other samples of this index value were lower. It should be noted that if the flow behavior index value shifted to 1, the Newtonian fluid behavior characteristic appeared, while this index value is closer to zero, indicating the rheological behavior characteristics of non‐Newtonian fluid (Burrell et al., 2010). Regarding the flow behavior and rheological properties of Doogh, it should be noted that other researchers have proposed Doogh (containing about 6% fat‐free dry matter) as Newtonian fluid. However, key parameters such as dry matter percentage, fat%, salt, homogeneity, and presence or absence of extracellular polysaccharides can affect the flow behavior and rheological characteristics of the Doogh samples. In addition, *Ostwald*‐*de Waele* model has been introduced by other researchers as a suitable mathematical model for predicting the flow behavior of products like Doogh and fermented milk beverages stabilized by hydrocolloids (Janhoj et al., 2008; Kiani et al., 2010; Koksoy & Kilic, 2004). Therefore, it was found that the presence of hydrocolloids in the Doogh formulation has changed the flow behavior of this product from Newtonian to non‐Newtonian.

**TABLE 5:** Rheological and statistical parameters of *Ostwald*‐*de Waele* model for traditional low‐fat Doogh prepared with different hydrocolloid compounds.

Storage temperature (°C)		F.G			Xanthan			Mix	
	*n*	*K* (Pa.s^n^)	*R* ^2^	*n*	*K* (Pa.s^n^)	*R* ^2^	*n*	*K* (Pa.s^n^)	*R* ^2^
5	0.3543	3.712	0.9870	0.3888	2.731	0.9989	0.5534	3.678	0.9654
10	0.3108	3.643	0.9865	0.3397	2.665	0.9943	0.4453	3.655	0.9467
15	0.3176	3.342	0.9892	0.2678	2.4987	0.9965	0.4543	3.698	0.9974
20	0.3165	3.189	0.9543	0.2567	2.4356	0.9678	0.4312	3.567	0.9964
25	0.2654	3.196	0.9753	0.2865	2.3786	0.9866	0.4531	3. 279	0.9659
30	0.2245	3.145	0.9775	0.2567	2.2296	0.9878	0.4219	3.186	0.9765

The flow behavior index (*n*) and stability index (*K*) have a direct relationship. For instance, in measuring the viscosity of different Doogh samples, it was observed that both these parameters exhibited a decreasing trend in viscosity with increasing storage temperature. As the temperature increased from 5°C to 30°C, the molecular movement speed enhanced in the samples. This changes the outcome of higher fluidity or declining stability index of Doogh samples. Additionally, the flow behavior index shifted from the non‐Newtonian power–law state toward the Newtonian state. In fact, the increasing temperature accelerates the molecular diffusion in the Doogh samples, which leads to product stability reduction and a shear‐thinning change to Newtonian behavior. The temperature shows an inverse relationship with both the flow behavior index and the stability index.

Arrhenius model (Equation 6) was used to determine the apparent viscosity relationship with temperature at different rotational speeds. Using linear regression, the prediction coefficient (*η∞*) and activation energy (*Ea*) were calculated, and their values for different hydrocolloids used in the low‐fat Doogh formulations are presented in Table 6. High values of coefficient of determination (*R* ^2^) indicate high viscosity correspondence of Arrhenius model (*R* ^2^ < 0.827). The results showed that increasing the rotational speed of all the hydrocolloid compounds used increased the activation energy. Also, the effect of different shear rates on regression equation changes Doogh samples observed in Table 7.

**TABLE 6:** Influence of rotational speeds on the activation energy of different traditional low‐fat Doogh formulations.

Type	Rotational speed (rpm)	Pre‐exponential coefficient^a^ (Pa.s)	Activation energy (kJ/Mol)	R2
	30	12.15	0.0027	0.992
F.G	50	12.06	0.0029	0.971
	100	11.65	0.0006	0.845
	200	18.19	0.000769	0.995
	30	13.78	0.0012	0.984
Xanthan	50	12.97	0.0013	0.965
	100	15.87	0.0001	0.997
	200	15.07	0.0002	0.094
	30	12.47	0.0035	0.97
Mix	50	12.32	0.0028	0.954
	100	13.76	0.0026	0.987
	200	19.15	0.00454	0.963

^a^ *η*_∞_: Arrhenius pre‐exponential coefficient or infinite apparent viscosity.

Mixture = 0.2% Fish gelatin + 0.2% xanthan.

**TABLE 7:** The effect of different share rates on regression equation changes in Doogh samples.

Type		Rotational speed (rpm)	Regression equation
		30	4.33 T^−1^ 1478 = lnη
	F.G	50	– 5.98T^−1^ 1455 = lnη
		100	7.54 – T^−1^ 1784 = lnη
		200	9.75 – T^−1^ 2189 = lnη
		30	6.755 – T^−1^ 1635 = lnη
	Xanthan	50	6.722 – T^−1^ 1552 = lnη
		100	8.661 – T^−1^ 2012 = lnη
		200	8.388 – T^−1^ 1810 = lnη
		30	5.89 – T^−1^ 1504 = lnη
	Mix	50	5.99 – T^−1^ 1485 = lnη
		100	6.43 – T^−1^ 1336 = lnη
		200	9.71 – T^−1^ 2307 = lnη

Doogh has an acidic nature and is similar to other acidified milk drinks, because of the aggregation of casein due to the low pH in this drink (Hashemi et al., 2015; Shirkhani et al., 2012).

The pH changes of different formulations of Doogh produced according to storage time are presented in Table 8. The results showed that the trend of these changes was in descending order of storage period. As it has been reported by several researchers, this increase could be due to the fermentation of lactose and production of lactic acid and other organic acids by lactic acid bacteria (LAB) (Haji Ghafarloo et al., 2020). These changes were lower for the control and Doogh samples containing gelatin. A considerable reduction of pH to the Doogh samples including fish gelatin, xanthan, and Mix was observed. The pH reduction value with the presence of the levels of gums was reported by Khodashenas and Jouki (2020). This phenomenon can be due to the presence of starters and hydrocolloids as appropriate additional nutrients that improve the viability of the starter bacteria. These factors supply better conditions for increasing culture growth. Ghosi Hoojaghanet et al. (2022) revealed that the addition of the tragacanth gum decreased the pH values of the experimental Doogh samples which is consistent with the results of this study (Ghosi Hoojaghanet et al., 2022).

**TABLE 8:** pH of low‐fat Doogh produced during storage time.

Type		1 day	7 day	14 day	21 day	30 day
	Control	4.35 ± 0.208^a^	4.26 ± 0.013^a^	4.14 ± 0.055^e^	4.11 ± 0.011^b^	4.07 ± 0.084^b^
	F.G	4.29 ± 0.0057^b^	4.25 ± 0.067^b^	4.17 ± 0.081^d^	4.15 ± 0.010^c^	4.12 ± 0.041^c^
	Xanthan	4.32 ± 0.016^b^	4.30 ± 0.010^a^	4.24 ± 0.032^a^	4.20 ± 0.056^a^	4.18 ± 0.057^a^
	Mix	4.31 ± 0.015^b^	4.28 ± 0.018^a^	4.20 ± 0.058^c^	4.17 ± 0.015^a^	4.15 ± 0.051^b^

Note: Values are given as mean with standard deviations (*n* = 3). Different letters in the same columns indicate significant differences between data (*p* < .05).

Furthermore, during the 30‐day storage period, this pH‐decreasing trend continued. The reason for the greater reduction in pH in the sample containing fish gelatin compared to other samples can be attributed to its structure and the presence of amino acids and suitable compounds that are readily utilized by microorganisms such as starter bacteria that lead to the production of secondary metabolites and lactic acid, which results in pH reduction. Also, in the sample containing xanthan, its unique and symmetrical structure made it less susceptible to microbial utilization. Additionally, the mixed sample benefited from the synergistic properties of hydrocolloids, which acted synergistic properties against starter culture and microbial flora growth that prevented changes in pH in the Doogh structure (Kamdem et al., 2020; Ranadheera et al., 2010; Williams & Phillips, 2021). Also, Solltani et al. (2012) pointed out that pH of Iranian drink (Doogh) was lower than 4.5. These results are in agreement with this project (Soltani et al., 2012).

The results of serum separation and organoleptic properties of low‐fat Doogh samples produced are presented in Tables 9, 10. In recent years, many researches have been published on the use of hydrocolloids as stabilizer agents which prevent phase separation in yoghurt drink formulations (Phillips & Williams, 2009; Shariati et al., 2020; Ziaolhagh & Jalali, 2017). As can be seen, adding hydrocolloid compounds decreased the serum separation of Doogh samples. According to the results, it was found that serum separation of low‐fat Doogh during a 30‐day storage period for control and Doogh samples containing gelatin, xanthan, and a combination of the two hydrocolloids were 19.78–45.07 mL, respectively. The fish gelatin and xanthan hydrocolloids in two single and combination forms used in this study, as food stabilizer, thickener, and binder, prevented serum separation in Doogh samples. It could be due to the ability of two hydrocolloids to enhance the viscosity and bind water. Therefore, water‐holding capacity of Doogh samples containing fish gelatin and xanthan improved.

**TABLE 9:** Serum separation of low‐fat Doogh produced during storage time.

Type	1 day	7 days	14 days	21 days	30 days
Control	19.78 ± 1.52^a^	23.98 ± 1.15^a^	32.09 ± 1.44^a^	35.32 ± 1.072^a^	45.07 ± 2.51^a^
F.G	0	1.08 ± 0.03^b^	2.21 ± 0. 55^b^	2.65 ± 0.033^b^	3.16 ± 0.11^b^
Xanthan	0	0	0.53 ± 0.12^c^	1.53 ± 0.55^bc^	1.62 ± 0.057^b^
Mix	0	0	0.03 ± 0.057^c^	0.56 ± 0.11^c^	0.84 ± 0.051^b^

Note. Values are given as mean with standard deviations (*n* = 3). Different letters in the same columns indicate significant differences between data (*p* < .05).

**TABLE 10:** Organoleptic properties of low‐fat Doogh produced during storage time.

Type		Color	Odor	Taste	Firmness	Overall acceptability
	Control	4.11 ± 0.10^b^	3.96 ± 0.045^b^	3.85 ± 0.11^c^	3.53 ± 0.084^c^	4.12 ± 0.054^c^
	F.G	4.33 ± 0.057^a^	4.5 ± 0.57^a^	4.27 ± 0.028^b^	4.36 ± 0.013^b^	4.31 ± 0.11^b^
	Xanthan	4.34 ± 0.053^a^	4.44 ± 0.010^a^	4.29 ± 0.072^b^	4.37 ± 0.057^b^	4.33 ± 0.15^b^
	Mix	4.37 ± 0.011^a^	4.32 ± 0.032^a^	4.46 ± 0.043^a^	4.52 ± 0.051^a^	4.58 ± 0.058^a^

Note. Values are given as mean with standard deviations (*n* = 3). Different letters in the same columns indicate significant differences between data (*p* < .05).

The synergistic effect of the combination of the two hydrocolloids on serum separation was higher than the gelatin and xanthan samples in a single form. According to the results of Table 9, it was found that in the case of gelatin hydrocolloids and their binary composition, no serum separation was observed after 7 days of storage. One of the most problems in producing acidic milk drinks is their two‐phase production and maintenance due to their low viscosity, low pH, and their effect on protein deposition (Azarikia & Abbasi, 2010). Basically, the stability of casein micelles at the natural pH of milk is due to the position of kappa‐caseins on the casein micelle surface, which prevents the micelles from approaching each other by forming hair layers on their surface and spatial and electrostatic repulsion mechanisms (Holland & Boland, 2014; Manguy & Shields, 2019).

On the other hand, under certain conditions, the hair layers become separated (broken down by milk‐clotting enzymes) or disintegrate (loss of effective network charge by decreasing pH, increasing ionic strength, and decreasing solubility), instability occurs in casein micelles. Because of the acidification of the medium, calcium phosphate gradually exits the micelles, the negative electric charge of the micelles decreases and the casein micelles disintegrate (Horne & Lucey, 2017). The mechanism of action of hydrocolloids depends on the molecular structure of the hydrocolloids used to prevent serum separation. Hence, it used pregnant through steric hindrance and electrostatic repulsion, the fermented beverages will likely stabilize the fermented beverages.

Results of sensory evaluation of Doogh samples showed a significant difference (*p* < .05) between the samples and the control sample for color, taste, firmness, odor, and overall acceptability (Table 10). According to the results, the highest amount of color was observed in the samples containing the binary combination of the two gums, which was not significantly different from those containing the gelatin and xanthan hydrocolloids. Therefore, the use of hydrocolloids did not affect the color of the samples. On the other hand, the lowest color belonged to the sample without hydrocolloid (control) and there was a significant difference between the samples and the others. Evaluations of odor characteristics showed a significant difference between the samples containing hydrocolloid compounds and the control sample, with the highest scores obtained for Doogh samples containing xanthan gum. The evaluation of the taste of Doogh samples showed that the samples containing the binary combination of these two hydrocolloids had the highest score. Similarly, Azarikia and Abbasi (2010) revealed that the presence of tragacanthin extracts significantly enhanced the taste score of Doogh. Also, the firmness of the samples showed a significant difference between the samples and the control sample. Studying average scores for firmness parameter determined the high desirability of Doogh samples enriched with hydrocolloids, so that the maximum amount of firmness was observed in the Mix sample. The results of total acceptability of samples also showed a significant difference between samples containing hydrocolloid and control sample. The highest score was obtained for the samples containing the binary combination of these two gums. The results also showed no significant difference between samples containing gelatin and xanthan hydrocolloids. The results of this study are consistent with Ziaolhagh and Jalali (2017). They reported that adding hydrocolloids such as xanthan gum could improve the sensory properties of bio‐Doogh (Ziaolhagh & Jalali, 2017).

## 4 CONCLUSION

The study of the rheological behavior of Doogh containing gelatin and xanthan hydrocolloids single and mixed form done using *Ostwald‐de Waele* model. The Mix sample had the lowest serum separation and highest overall acceptability during storage time. On the other hand, pH changes revealed that pH control sample was lower than gelatin and xanthan samples after 30‐day production. Also, Mix sample had higher shear stresses at the same temperature and constant shear rates than the samples including xanthan and gelatin in a single form. Also, in all samples containing hydrocolloids, quickly decreasing apparent viscosity occurred at low shear rates, whereas after this severe decrease, the apparent viscosity of the samples decreased with fewer slopes. Various factors such as dry matter, fat, salt, homogeneity, and presence or absence of hydrocolloids can affect the flow behavior and rheological properties of Doogh. Considering the health effects of Iranian drink (Doogh), further research is needed to investigate the factors relating to the rheological properties of the Doogh, which can affect the phase separation problem and product‐friendly market.

## AUTHOR CONTRIBUTIONS

**Fatemeh Rahmati:** Formal analysis (equal); writing – original draft (equal). **Abbas Mahjoorian:** Data curation (equal); formal analysis (equal); methodology (equal); writing – review and editing (equal). **Fatemeh Fazeli:** Writing – review and editing (equal). **Sharagim Ranjbar:** Software (equal); writing – review and editing (equal).

## CONFLICT OF INTEREST STATEMENT

The authors declared that there is no conflict of interest.

## ETHICS STATEMENT

This article does not contain any studies with human participants or animals performed by any of the authors.

## INFORMED CONSENT

For this type of study, formal consent is not required.

## CONSENT TO PARTICIPATE

Corresponding and all the coauthors are willing to participate in this manuscript.

## Data Availability

Even though adequate data has been given in the form of tables and figures, however, all authors declare that if more data is required then the data will be provided on a request basis.
